# *Candida glabrata* Binding to *Candida albicans* Hyphae Enables Its Development in Oropharyngeal Candidiasis

**DOI:** 10.1371/journal.ppat.1005522

**Published:** 2016-03-30

**Authors:** Swetha Tati, Peter Davidow, Andrew McCall, Elizabeth Hwang-Wong, Isolde G. Rojas, Brendan Cormack, Mira Edgerton

**Affiliations:** 1 Department of Oral Biology, School of Dental Medicine, University at Buffalo, Buffalo, New York, United States of America; 2 Department of Molecular Biology and Genetics, Johns Hopkins University, Baltimore, Maryland, United States of America; Louisiana State University Health Sciences Center, UNITED STATES

## Abstract

Pathogenic mechanisms of *Candida glabrata* in oral candidiasis, especially because of its inability to form hyphae, are understudied. Since both *Candida albicans* and *C*. *glabrata* are frequently co-isolated in oropharyngeal candidiasis (OPC), we examined their co-adhesion *in vitro* and observed adhesion of *C*. *glabrata* only to *C*. *albicans* hyphae microscopically. Mice were infected sublingually with *C*. *albicans* or *C*. *glabrata* individually, or with both species concurrently, to study their ability to cause OPC. Infection with *C*. *glabrata* alone resulted in negligible infection of tongues; however, colonization by *C*. *glabrata* was increased by co-infection or a pre-established infection with *C*. *albicans*. Furthermore, *C*. *glabrata* required *C*. *albicans* for colonization of tongues, since decreasing *C*. *albicans* burden with fluconazole also reduced *C*. *glabrata*. *C*. *albicans* hyphal wall adhesins Als1 and Als3 were important for *in vitro* adhesion of *C*. *glabrata* and to establish OPC. *C*. *glabrata* cell wall protein coding genes *EPA8*, *EPA19*, *AWP2*, *AWP7*, and *CAGL0F00181* were implicated in mediating adhesion to *C*. *albicans* hyphae and remarkably, their expression was induced by incubation with germinated *C*. *albicans*. Thus, we found a near essential requirement for the presence of *C*. *albicans* for both initial colonization and establishment of OPC infection by *C*. *glabrata*.

## Author Summary

Understanding how *Candida glabrata* is able to establish oral mucosal infections is particularly important since many *C*. *glabrata* strains are innately resistant to azole antifungal drugs used in treating mucosal and disseminated infections. The epidemiology of *C*. *glabrata* oral infections shows that *C*. *glabrata* is very often present as a co-infection with *Candida albicans*. Here we suggest a mechanism to explain this clinical finding. We show that *C*. *glabrata* is unable to colonize the oral mucosa in a murine oral infection model. However, prior or co-colonization by *C*. *albicans* allows *C*. *glabrata* to colonize and persist in the oral cavity. Mechanistically, we show that *C*. *glabrata* binds specifically to *C*. *albicans* hyphae, mediated by hyphally expressed *ALS* adhesins in *C*. *albicans* and cell surface proteins in *C*. *glabrata* that are transcriptionally up-regulated in the presence of *C*. *abicans*. In this sense, *C*. *glabrata* is a piggy-back fungus that relies upon binding to *C*. *albicans* hyphae for oral colonization. This finding has implications for treatment of oral candidiasis and may shed light on colonization mechanisms of other non-hyphae producing fungi.

## Introduction

Oropharyngeal candidiasis (OPC) is an opportunistic mucosal infection caused by *Candida* species [1,2]. *Candida albicans* and *Candida glabrata* are the first and second major etiological agents of OPC, respectively [3]. Although other *Candida* species, including *C*. *parapsilosis*, *C*. *tropicalis*, and *C*. *krusei*, may be isolated as the sole species from oral infection sites, single species infection by *C*. *glabrata* alone is rare [4,5]. *C*. *glabrata* is most frequently co-isolated along with *C*. *albicans* in mixed species oral infections [4,6,7]. Oral infections involving *C*. *glabrata* have increased by 17% over the past several years [7], and are particularly common in cancer patients, denture-wearers, or following prolonged use of broad spectrum antibiotics, steriods or following head and neck radiation therapy [3]. These infections were often associated with multiple *Candida* species [3,4]. Oral infections with mixed *C*. *albicans* and *C*. *glabrata* were found to be more severe and difficult to treat [5] since many *C*. *glabrata* strains are innately resistant to azole antifungal agents used in treating mucosal infections. Prophylactic use of azole antifungal drugs has been implicated as a major cause for the increase in non-*C*. *albicans* fungemia [8]. Fungemia caused by *C*. *glabrata* has high mortality especially in adult patients in intensive care units [9], and although fluconazole prophylaxis has reduced the incidence of invasive candidiasis in high-risk neonates and immunosuppressed patients, there has been little effect on the overall incidence of *C*. *glabrata* candidiasis. Given the frequency of *C*. *glabrata and C*. *albicans* co-infection, it is imperative to understand the mechanisms deployed by *C*. *glabrata* in co- infections with *C*. *albicans*.

*C*. *albicans* is a diploid, polymorphic fungus that exists in yeast, hyphal, and psuedohyphal forms [10]. *C*. *albicans* hyphae express numerous proteins that enhance virulence by adhering to host cells or damaging host tissue [11]. *C*. *albicans* hyphae are known to penetrate epithelial surfaces, damage endothelial cells, and aid in systemic infection by colonizing different organs such as kidneys, spleen and brain [10,12]. Als (Agglutinin Like Sequence proteins), Hwp1p (Hyphal wall protein), and Eap1 (Enhanced Adherence to Polystyrene) are well-characterized *C*. *albicans* hyphal wall adhesins that mediate *C*. *albicans* interaction with host epithelial, endothelial and host tissue proteins [13–15]. *C*. *albicans* adhesins contribute not only to its ability to adhere and colonize multiple types of host tissues, but also serve as binding moieties for other microbes such as *Streptococcus gordonii*, *Pseudomonas aeruginosa*, and *Staphylococcus aureus* [16–19]. It is therefore possible that one or more *C*. *albicans* hyphal-specific adhesins may play a role in *C*. *glabrata* interaction as well.

In terms of host tissue invasion, *C*. *albicans* has a fitness advantage over *C*. *glabrata* in terms of its ability to switch between yeast to hyphal forms. By contrast, *C*. *glabrata* virulence must be independent of its morphology, since it lacks the ability to form true hyphae. However, *C*. *glabrata* is likely to express specific adhesins in order to establish colonization [20,21]. Phylogenetic analysis of the *C*. *glabrata* genome showed 66 putative cell wall proteins, of which only a few have been well characterized in terms of host cell adhesion [13]. Cell wall protein families known to be involved in adhesion to endothelial and epithelial cells include the *EPA* (Epithelial cell adhesin), *AED* (Adherence to endothelial cells), and *PWP* (PA14 domain containing Wall Protein) proteins [13]. *C*. *glabrata* Epa1, 6, and 7 adhesins bind to both endothelial and epithelial host cells [22,23], while Pwp7p and Aed1p are known to interact with endothelial cells [13]. Deletion of these Epa1 adhesins attenuated virulence in a murine model of disseminated candidiasis [22,23]. The role of *C*. *glabrata* adhesins, beyond their ability to mediate adherence to host tissues, is understudied. We hypothesize that one or more of these adhesins may promote interspecies interaction with *C*. *albicans* during mixed species OPC.

Co-adhesion is the basis for both single and multispecies colonization in the host [24]. Co-adhesion in bacteria is well studied and it has been established that the expression of multiple bacterial adhesins drive interspecies oral bacterial colonization [24,25]. Although mixed infections of *C*. *glabrata* and *C*. *albicans* occur frequently, the mechanism of co-adhesion and interspecies colonization is not well understood [4]. In our study, in spite of *C*. *glabrata* encoding several cell wall adhesins known to bind host epithelial and endothelial cells, we documented poor colonization in our murine OPC model. We hypothesized that co- infection or prior infection with *C*. *albicans* may facilitate *C*. *glabrata* infection. Here we characterize the co-colonization of *C*. *glabrata* and *C*. *albicans* in a murine model of OPC, and explore the role of cell wall proteins from both species in mediating cell-cell interaction and co-colonization.

## Results

### *C*. *glabrata* and *C*. *albicans* show enhanced growth in dual species biofilm

**Fig 1:**
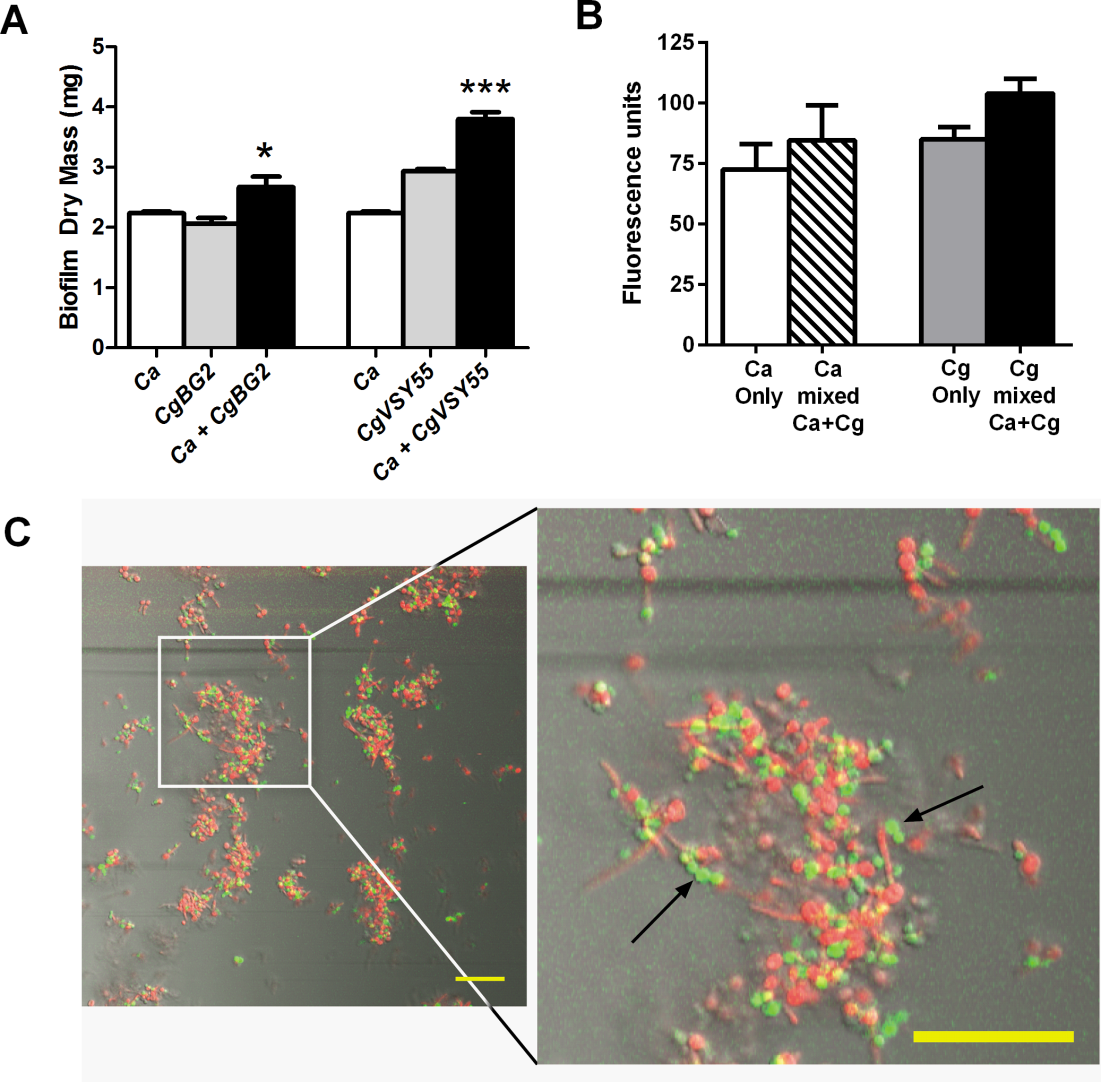
*C*. *glabrata* and *C*. *albicans* dual species biofilms showed increased biofilm mass that reflects C. glabrata adherence to C. albicans hyphae. (**A**) *C*. *albicans CAI4* and *C*. *glabrata* strains *CgBG2* and *CgVSY55* were grown as biofilms for 24h in plastic wells. Both *CgBG2* and *CgVSY562* strains showed significantly (*P<0.02, ***P<0.001 by student’s t test) increased biomass in a dual species biofilm (black bars) as compared to single species biofilm (white and grey bars). Fluorescent quantitation of co-culture of *C*. *albicans CAF2-ycherry* with *C*. *glabrata* CgVSY55 showed enhanced growth of both species compared with single species (Fig 1B). When both species were co-cultured under dynamic flow conditions, *C*. *glabrata* (green) was able to bind *C*. *albicans* (red) biofilms and hyphae under conditions of flow (Fig 1C). Fluorescent images were merged with DIC images to enhance hyphal visibility. Hyphal binding is indicated by arrows. Scale bars represent 50 μm.

We initially performed an *in vitro* biofilm assays to test whether *C*. *albicans* and *C*. *glabrata* have any cooperative growth effects. Two strains of C. *glabrata* (*BG2* wild type, WT) and a GFP-expressing strain *CgVSY55* (*ura3Δ*::*hph* ScPGKp-yEGFP-URA3-CEN-ARS) derived from a *CgDSY562* WT [26] and two strains of *C*. *albicans* (*CAI4* WT with URA replaced, URA+) or *CAF2-yCherry* strain [27] were used in biofilm experiments. In a static plate assay, *C*. *albicans CAI4* and *CgBG2* each formed single species biofilms with similar robustness. However, when grown together as a dual species biofilm, the total dry weight was significantly (P<0.001) higher compared to single species (Fig 1A). Fluorescent quantitation of co-culture of *C*. *albicans CAF2-yCherry* with *C*. *glabrata* CgVSY55 under static biofilm growth showed enhanced growth of both species occurred compared with single species (Fig 1B). In contrast, under dynamic flow conditions, *C*. *glabrata* (*CgVSY55)* alone was unable to form biofilms within the flow chamber, while *C*. *albicans* formed abundant biofilms. However, when both species were co-cultured under dynamic flow conditions, *C*. *glabrata* CgVSY55 cells (green) were found associated with *C*. *albicans* (red) nascent biofilm regions, and were concentrated along *C*. *albicans* hyphae (Fig 1C arrows).

### *C*. *glabrata* adheres to *C*. *albicans* hyphae

**Fig 2:**
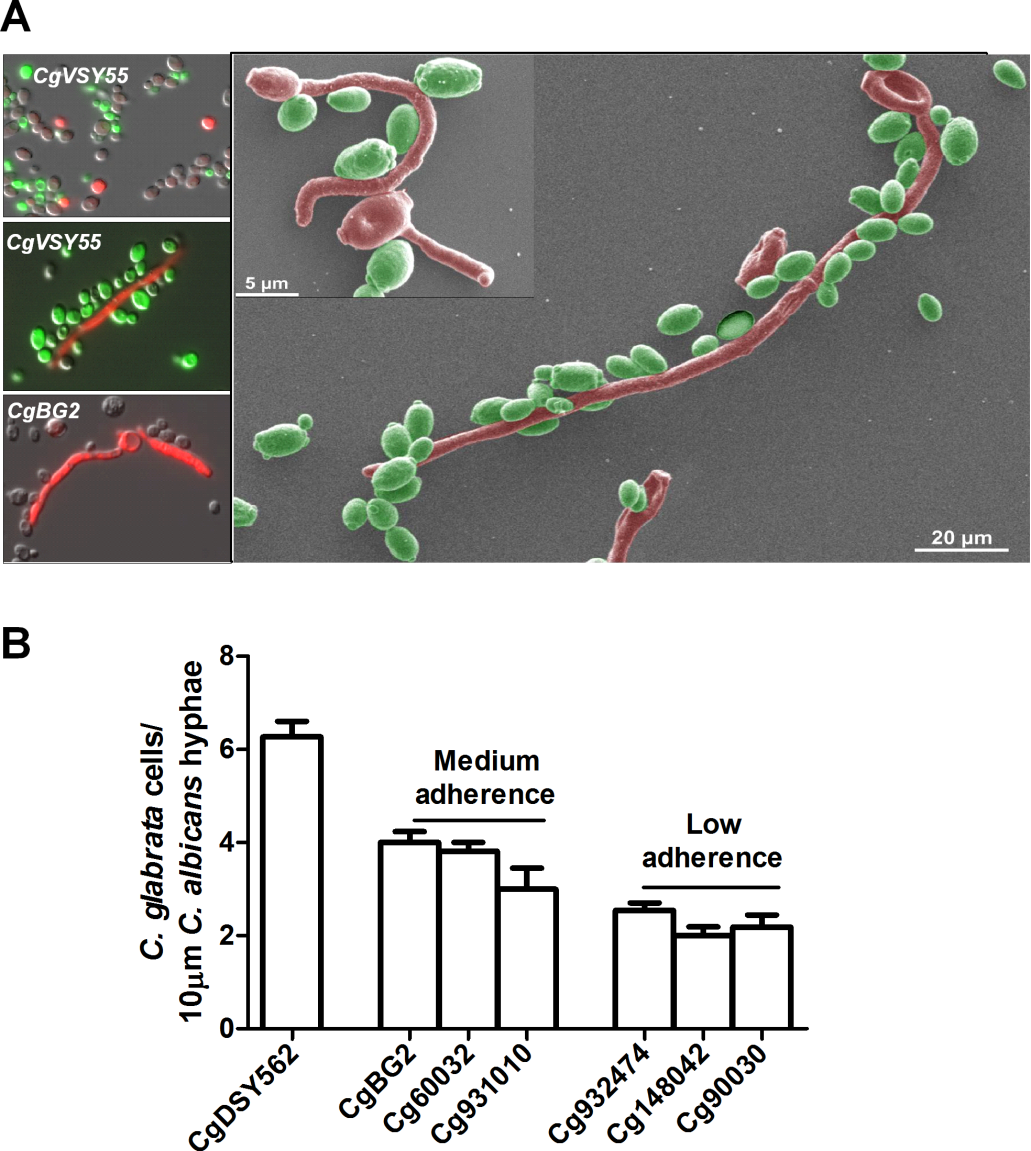
*C*. *glabrata* adheres to *C*. *albicans* hyphae. *C*. *albicans* germinated or yeast cells were incubated with *C*. *glabrata* cells at 1:1 ratio for 60 min. While little adherence of *C*. *glabrata* was found with *C*. *albicans* yeast, large numbers of *C*. *glabrata CgVSY55* or *CgBG2* were found adherent along the length of *C*. *albicans* hyphae using fluorescence microscopy (**A** left panel). Adhesion of along the length of *C*. *albicans* hyphae was also found in scanning electron micrograph images of *CgVSY55* and *CAF2-yCherry* (**A** right panel). Magnifications are 500X (scale bar: 5μm, 20μm). C. *glabrata* cells adhesion to *C*. *albicans* hyphae (10μm length) was quantitated microscopically. Seven wild type strains of *C*. *glabrata* were screened for adherence to *C*. *albicans* hyphae and grouped as high (*CgDSY562*), medium (*CgBG2*, *Cg60032*, *Cg931010*) and low (*Cg932474*, *Cg148042*, *Cg90030*) binders. (**B**).

To further examine how these two *Candida* species might be interacting, we examined their association directly by fluorescence microscopy. *C*. *albicans* cells were grown in YNB + 1.25% glucose (for yeast phase cells) or in YNB + 1.25% N-acetyl glucosamine at 37°C (to induce hyphal cells) for 3 h. *C*. *albicans* cells were then incubated with *C*. *glabrata* cells at 1:1 ratio for 60 min. *C*. *glabrata* cells did not adhere with *C*. *albicans* yeast cells (Fig 2A, upper left); however they showed strong adhesion along the length of germinated *C*. *albicans* hyphae (Fig 2A left). Scanning Electron Microscopy (SEM) further illustrated this interaction showing that *C*. *glabrata* cells adhered along the entire length of *C*. *albicans* hyphae (Fig 2A right). We observed that *C*. *glabrata* cells formed rows of adherent cells along the length of hyphae, but did not adhere to other *C*. *glabrata* cells. Next, we quantified adhesion as defined by the number of *C*. *glabrata* cells adhering to 10 μm length of *C*. *albicans* hyphae in seven different strains of *C*. *glabrata*. Among the *C*. *glabrata* strains examined, *CgDSY56* (the parent strain of *CgVSY55*) had significantly (P<0.0001) higher adherence (6.4 ± 0.2 cells / 10 μm hyphae, high adherence strain) when compared to other strains tested. *CgBG2* and *Cg960032* (4.2 ± 0.2 cells / 10 μm hyphae) showed medium adherence; and *Cg931010*, *Cg932474*, *Cg148042*, and *Cg90030* showed low adherence (3.0 ± 0.2cells / 10 μm hyphae) (Fig 2B).

Yeast to hyphae transition in *C*. *albicans* induces expression of hyphal-specific proteins as well as altering mannans and glucans levels in the hyphal cell wall [28]. To identify whether binding between *C*. *albicans* and *C*. *glabrata* was mediated by cell wall carbohydrates, we performed blocking experiments with *C*. *albicans* using concanavalin A (which binds cell wall mannans) and an antibody to β,1–3 glucan at concentrations that we previously showed provided good cell coverage [29]. *C*. *albicans* hyphae were treated with concanavalin A or with β,1–3 glucan Ab for 30 min, washed, then incubated with *C*. *glabrata*; however *C*. *glabrata* adhesion to *C*. *albicans* hyphae was unchanged, suggesting that *C*. *albicans* adhesion is not mediated by binding to *C*. *albicans* mannose or β,1–3 glucan. This is consistent with the fact that we did not detect *C*. *glabrata* binding to other *C*. *glabrata* cells since the *C*. *glabrata* cell wall contains both mannan and β,1–3 glucan.

### *C*. *albicans* hyphal wall Als adhesins are needed for *C*. *glabrata* adherence

**Fig 3:**
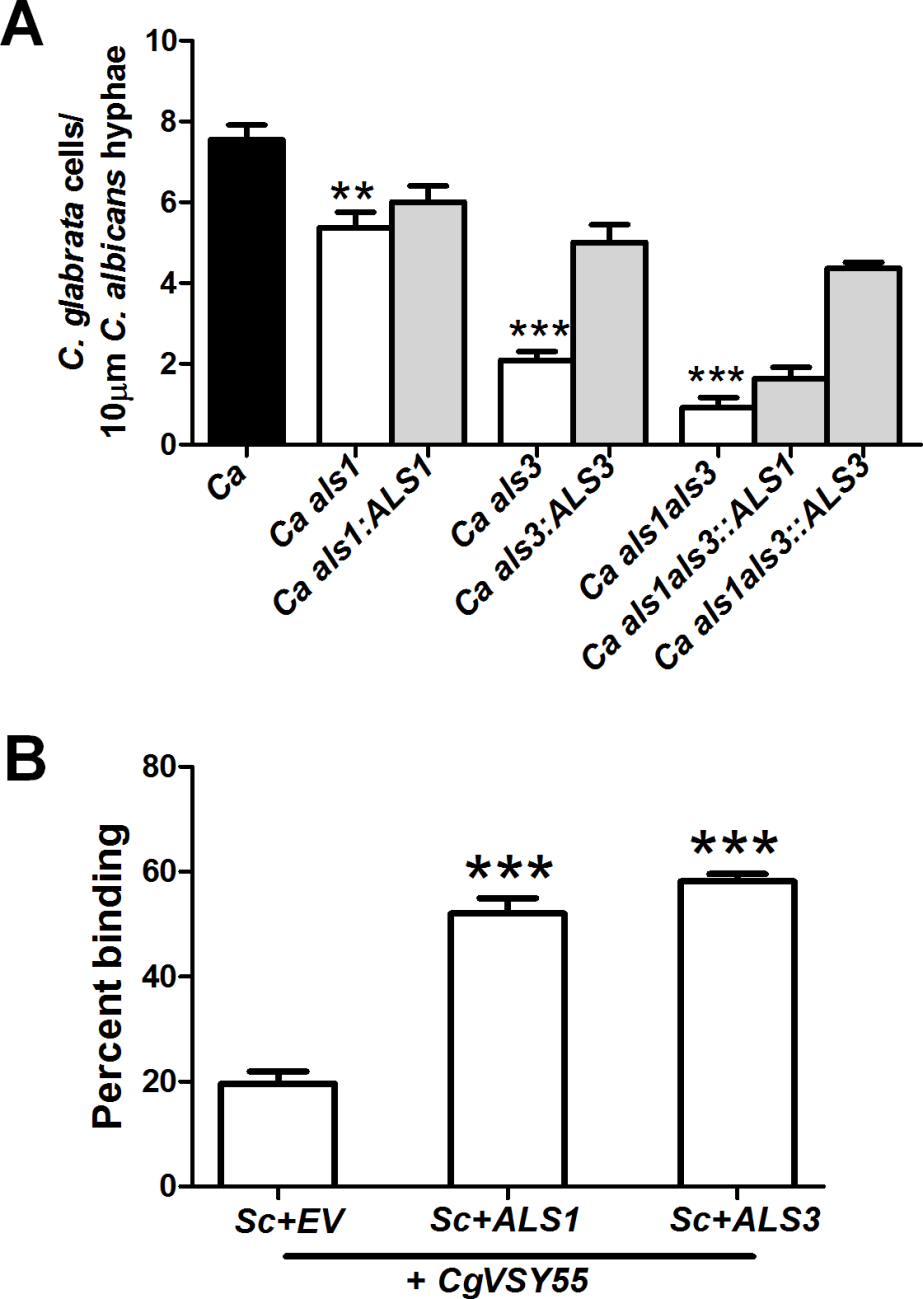
C. albicans Als3 and Als1 hyphal cell wall adhesins are involved in C. glabrata adherence to C. albicans hyphae. (**A**) *C*. *glabrata (CgVSY55)* adherence to *C*. *albicans* Als1, Als3 and Als1/3 deficient strains was measured using fluorescent microscopy. *C*. *glabrata* showed a significant (**P<0.001,***P<0.0006) decrease in adherence to *C*. *albicans* hyphae of *als3Δ/Δ*, *als1Δ/Δ*, and als1/*als3Δ/Δ* mutants; while *C*. *albicans* Als1 and Als3 complementation strains had restored adherence. (**B**) *S*. *cerevisiae* strains expressing *C*. *albicans* Als1 and Als3 adhesins and an empty vector (control) were incubated with *CgVSY55* and their adherence was quantifed by direct visualization. Both Als1 and Als3 expressing *S*. *cerevisiae* strains had a significant (***P<0.001) increase in Binding Index compared to control empty vector. Differences between groups were analyzed by a student’s t test.

We hypothesized that *C*. *glabrata* might bind *C*. *albicans* cell wall proteins directly. To test candidate *C*. *albicans* hyphal wall adhesins required for *C*. *glabrata* adhesion, we performed co-adhesion assays with *ALS1* and *ALS3* deficient *C*. *albicans* (Fig 3). We found that *C*. *glabrata* had significantly decreased adherence to hyphae of a *C*. *albicans als3Δ/Δ* mutant (72.3% reduction), an *als1Δ/Δ* mutant (28.8% reduction), and an *als1*/*als3Δ/Δ* double mutant (86% reduction). *ALS1* and *ALS3* complementation strains showed restoration of adherence to levels closer to that of wild type strain (Fig 3A). To further validate the role of *C*. *albicans* Als1 and Als3, we performed a quantitative adherence assay by direct microscopic observation using S. *cerevisiae* strains expressing *C*. *albicans ALS1* and *ALS3* with a GFP-tagged *C*. *glabrata* strain. Both Als1 and Als3 expressing S. *cerevisiae* strains showed significantly higher binding (Binding Index = 52.0 ± 3.0 and 58.2 ± 1.4, respectively) compared to *S*. *cerevisiae* expressing an empty vector (Binding Index = 19.7 ± 2.3) (Fig 3B).

### *C*. *albicans* is required to establish *C*. *glabrata* infection in OPC

**Fig 4:**
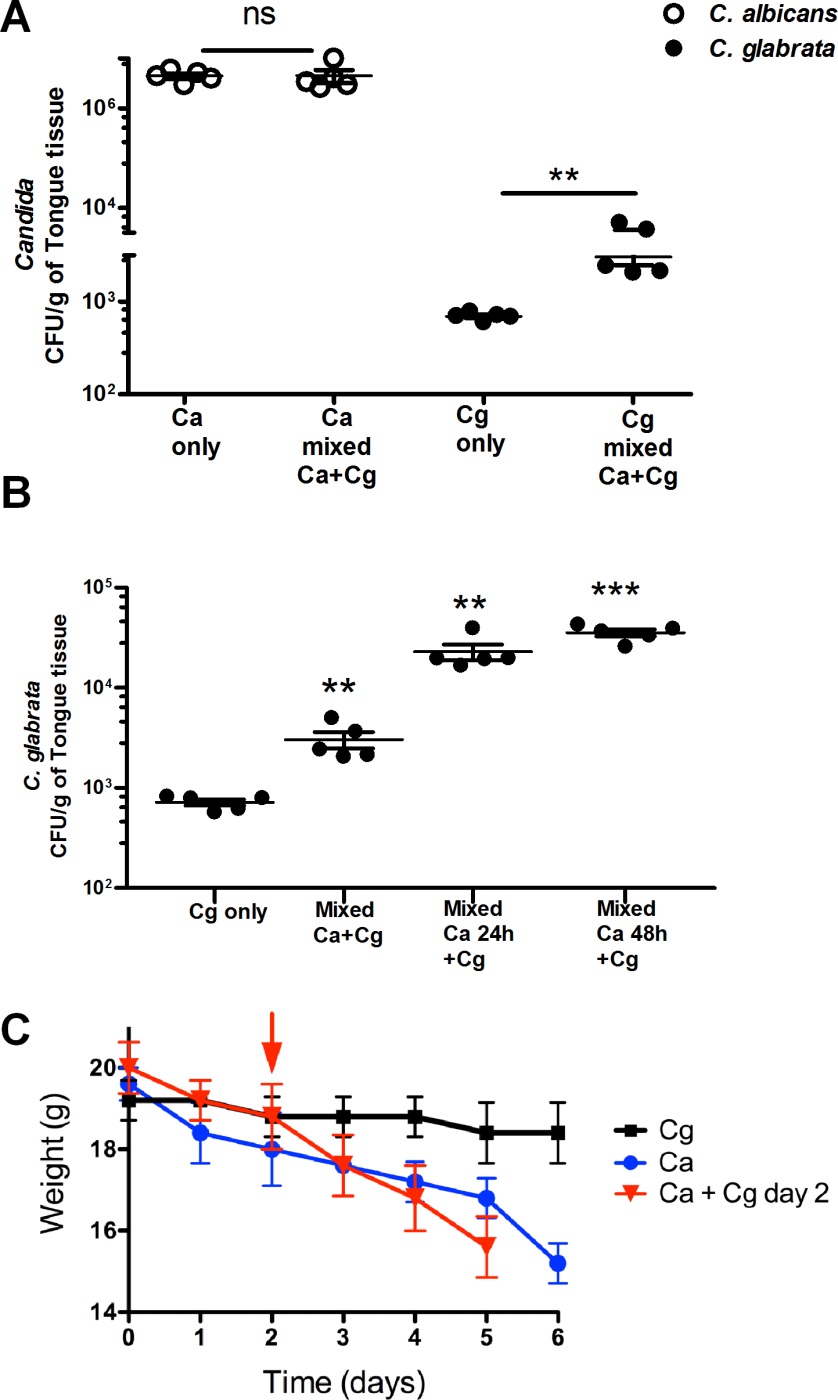
C. albicans enhances C. glabrata colonization in murine oropharyngeal candidiasis model. (**A**) Colony Forming Units (CFU) per gram of tongue tissues were recovered from infections with *C*. *albicans* alone, *C*. *glabrata* alone or *C*. *albicans* and *C*. *glabrata* co-infected mice after 5 days. *C*. *glabrata* colonization increased by one log-fold upon co-infection with *C*. *albicans*. (**B**) Pre-establishing *C*. *albicans* infection (24 h or 48 h before *C*. *glabrata* infection) further increased subsequent *C*. *glabrata* colonization by two log-fold in mice tongue tissues. Differences between groups were analyzed by a student’s t test (**P<0.003, **P<0.001, ***P<0.0001). (**C**) *C*. *glabrata* infection alone did not result in mice weight loss, however the rate of weight loss was accelerated in the mixed infection (red arrow indicates initiation of *C*. *glabrata* infection). Animal weights (mean and SD of each group, n = 7) are shown for each group (black, *C*. *glabrata* infection only; blue *C*. *albicans* infection only; red, *C*. *albicans* infection for 48 h followed by *C*. *glabrata* infection).

To determine whether our observed binding between *C*. *albicans* and *C*. *glabrata* has relevance *in vivo*, we examined the ability of *C*. *glabrata* to establish infection in our murine model of OPC. Since *C*. *glabrata* has not been used before in OPC infection models, we began with a single species oral infection of C57BL/6 mice with *C*. *glabrata* alone as we have previously done with *C*. *albicans* (Fig 4A). In this model, sublingual infection with a *C*. *albicans* inoculum of 1 X 10^6^ cells/ml typically produces clinical symptoms and white tongue plaques 4–5 days post infection, and recovery of 1 X 10^7^ CFU / gm tongue tissue at 5 days post infection. Surprisingly, in no infection experiments using *C*. *glabrata* did we observe the typical appearance of white tongue plaques indicative of clinical infection. We tried varying immunosuppressive agents (triampicinolone acetonide, cyclophosphamide), mouse strains (BALB/c, IL17RAk/o) and used inocula size of *C*. *glabrata* ranging from 1 X 10^7^ to 1 X 10^10^ cells/ml. In all cases, infection with *C*. *glabrata* alone resulted in no clinical appearance of disease or weight loss in animals. Consistent with this lack of disease, the recoverable *C*. *glabrata* CFUs from the tongue were extremely low (4–7 X 10^2^ CFU/g of tongue tissue).

Since our *in vitro* biofilm and adhesion assays showed enhanced adhesion and growth of *C*. *glabrata* when mixed with *C*. *albicans*, we next attempted a mixed infection with *C*. *glabrata* (1X10^9^ cells/ml) either as a co-infection with *C*. *albicans* (5 X 10^7^ cells/ml); or as a delayed infection with *C*. *glabrata* 24 or 48 h after infection of *C*. *albicans* (Fig 4). *C*. *glabrata* CFU were significantly (P<0.02) increased by ten-fold (3 X 10^3^ CFU/g of tongue tissue) when mice were co-infected with *C*. *albicans* (Fig 4A). Co-infection with *C*. *glabrata* did not alter *C*. *albicans* infection levels (1.2 X 10^7^ CFU/g of tongue tissue) compared with *C*. *albicans* infection alone (Fig 4A). However, delaying *C*. *glabrata* infection for 24 or 48 h after establishment of *C*. *albicans* infection further increased *C*. *glabrata* oral infection by a further 10-fold (3.5–4.5 X1 0^4^ CFU/g of tongue tissue, P<0.0001) compared to *C*. *glabrata* single species infection (Fig 4B). Mean animal weights did not change upon *C*. *glabrata* infection only (Fig 4C). However, mice lost weight more rapidly following a mixed infection compared with infection by *C*. *albicans* alone, so that mice in the mixed infection group had to be sacrified one day sooner due to total weight loss compared with mice infected with *C*. *albicans* only (Fig 4C). Thus our data show that levels of oral infection of *C*. *glabrata* were signifcantly increased by an established *C*. *albicans* oral infection and the rate of weight loss was increased upon dual species infection.

**Fig 5:**
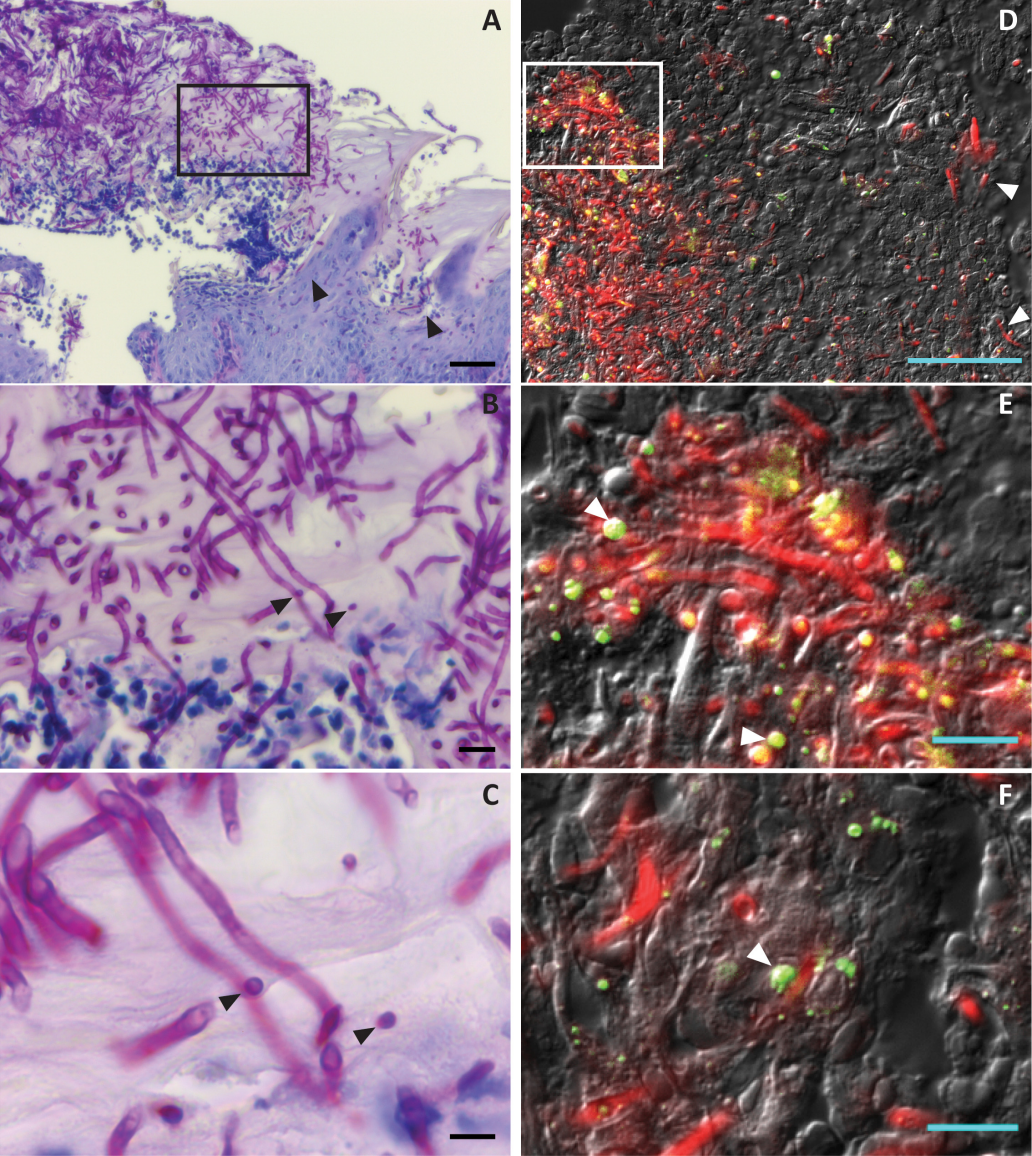
C. albicans and C. glabrata are co-localized and both invade murine tongue tissues in a mixed infection. (**A, B** and **C**) PAS stained formalin-fixed and paraffin embedded sections (5 μm) from tongues of mixed *C*. *albicans* and *C*. *glabrata* infection at day 5 showed widespread fungal plaques and hyphal invasion (yeast are stained magenta) of superficial epithelium (box) and underlying epithelium (arrows). Dark blue cells are neutrophils, lighter blue cells are tongue epithelia. Magnification is 10x. (**B**) 40x and (**C**) 100x magnification show *C*. *albicans* hyphae with associated yeast cells within the mucosa. (**D, E** and **F**) Immunofluorescent and DIC merged images of *C*. *albicans* (red) and *C*. *glabrata* (green) from tongues at day 5 post-infection. (**D)** Arrowheads show hyphae penetration into the epithelium. (**E** and **F**) Arrowheads at left indicate *C*. *glabrata* in contact with hyphae, while other *C*. *glabrata* are within epithelium unassociated with *C*. *albicans* hyphae (**E**, lower arrow). Scale bars represent, in order (A-F), 50, 10, 5, 50, 10, and 10 μm.

Next, we examined tongues of mixed-infected mice histologically to determine whether *C*. *glabrata* alters *C*. *albicans* invasive properties and to identify the localization of *C*. *glabrata* infection within the mucosal epithelium. For these experiments we infected mice with fluorescent-tagged strains of *C*. *albicans* (*CAF2-yCherry*) on day 0 and *C*. *glabrata* (*CgVSY55*) on day 2; and collected tongue tissues on day 5. Tongues were sectioned and stained with either PAS to visualize fungal-tissue architecture or cryo-sectioned for visualization of yeast cell localization by fluorescence microscopy. Tongues from mice with mixed infection showed robust fungal plaque formation as well as extensive *C*. *albicans* hyphal penetration of the superficial epithelium (Fig 5A, boxed region) as well as invasion into some regions of the underlying epithelium and lamina propria (Fig 5A, arrows). Closer inspection of these regions showed widespread *C*. *albicans* hyphae; and in some areas yeast cells were observed both adherent to hyphae and as unattached cells that were likely to be *C*. *glabrata* (Fig 5B and 5C, arrows). Fluorescent imaging of these regions confirmed that the majority of tissue invasion was with *C*. *albicans* hyphae (Fig 5D, boxed region, red), however *C*. *glabrata* cells (green) were also observed within these tissues both associated with *C*. *albicans* hyphae as well as being unconnected and separate within the epithelium (Fig 5E and 5F, arrows). In contrast, mono-species *C*. *glabrata* infection resulted on only very small superficial plaques that were localized on the surface mucosa without any invasion. Thus, infection of oral epithelium with *C*. *albicans* and the presence of its hyphae were permissive for infection and tissue invasion by *C*. *glabrata*.

**Fig 6:**
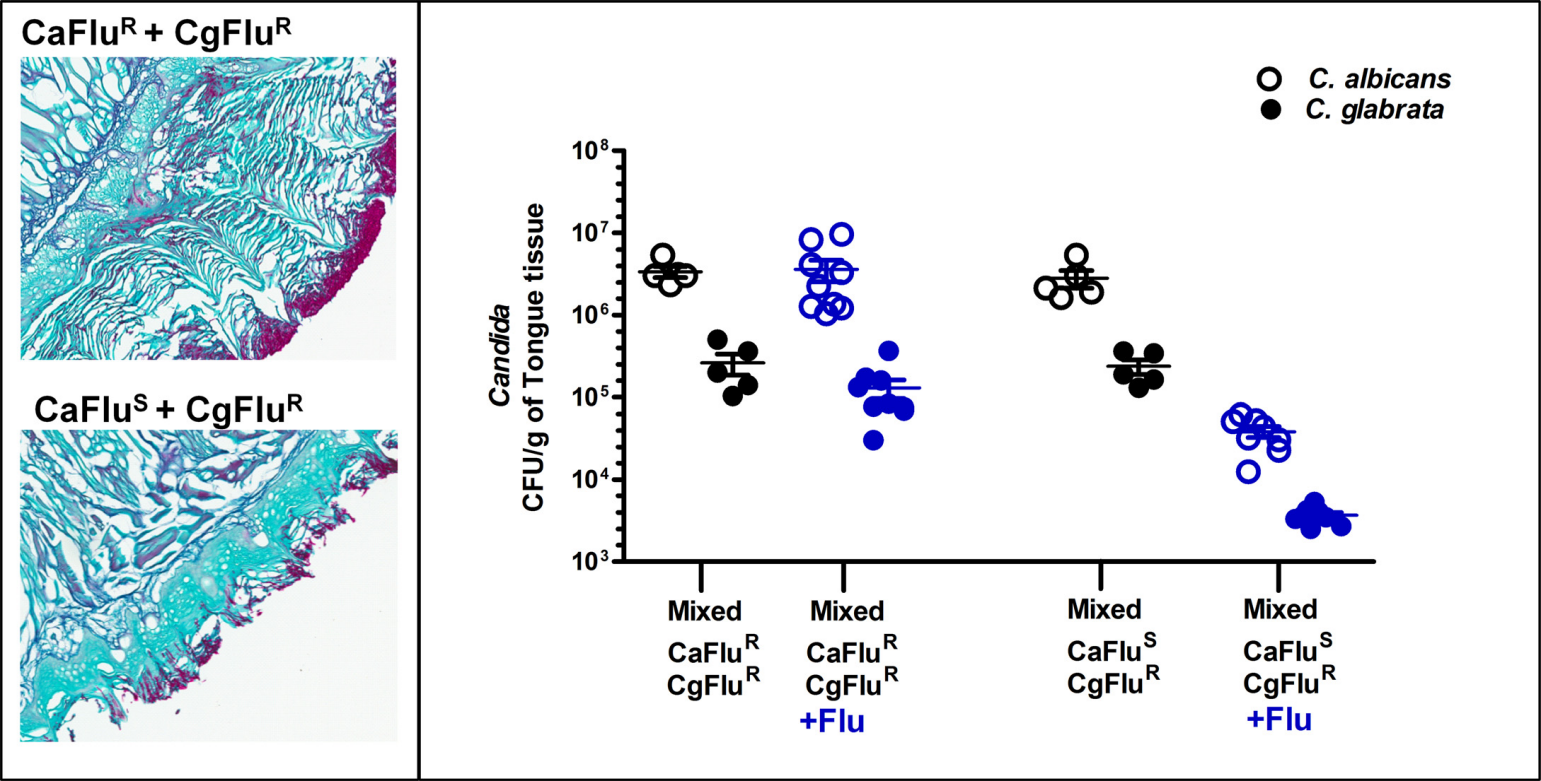
C. glabrata requires C. albicans for early infection. Mixed species oral infection with fluconazole resistant (*CaFlu* ^*R*^, *CgFlu* ^*R*^) and fluconazole sensitive (*CaFlu* ^*S*^) strains showed a significant reduction in number *C*. *glabrata* fluconazole resistant *CgFlu* ^*R*^ cells (solid circles) following fluconazole treatment (despite being fluconazole resistant) that was proportional with reduction in fluconazole sensitive *C*. *albicans CaFlu* ^*S*^ (open circles) CFUs (**right**). Tongue tissues were stained with Periodic acid-Schiff stain and viewed at 10X magnification (**left**). *CaFlu* ^*S*^ and *CgFlu* ^*R*^ infected mice showed normal tongue histology with a reduced fungal burden following fluconazole treatment, while *CaFlu* ^*R*^ and *CgFlu* ^*R*^ (yeast cells are shown in pink) infected mice showed typical hyphal invasion of the superficial epithelium with high fungal burden.

To further confirm the requirement of *C*. *albicans* for *C*. *glabrata* for initial infection, we treated mice with fluconazole (Flu) after establishing mixed infection using Flu sensitive *(CaFlu* ^*S*^) or Flu resistant *C*. *albicans (CaFlu* ^*R*^) strains and Flu resistant *C*. *glabrata (CgFlu* ^*R*^) (Fig 6). Mice were treated with Flu for four days after an oral mixed infection was already established for four days. As expected, Flu treatment did not alter infection levels of either species in a mixed infection with *CgFlu* ^*R*^ and *CaFlu* ^*R*^ strains. However, for a mixed infection with *C*. *glabrata CgFlu* ^*R*^ and *C*. *albicans CaFlu* ^*S*^ strains, Flu treatment resulted in significant (by two logs, P<0.001) reduction of both *C*. *glabrata* and *C*. *albicans*. Flu treated animals infected with *CaFlu* ^*R*^ strains in a mixed infection lost significantly (P<0.05) more weight (21.2 ± 0.2%) than mice infected with *C*. *albicans CaFlu* ^*S*^ strains (18.9 ± 0.3%). Although we could not determine the co-locallization of *C*. *albicans* and *C*. *glabrata* histologically due to lack of fluorescent markers in Flu resistant strains, examination of tongues confirmed the reduction in superficial epithelial fungal burden and invasion upon Flu treatment (Fig 6). Thus, *C*. *glabrata* infection levels were proportional to those of *C*. *albicans*, showing that *C*. *glabrata* requires the presence of *C*. *albicans* for early infection *in vivo*.

### *C*. *glabrata* requires *C*. *albicans* Als1 and Als3 adhesins in OPC

**Fig 7:**
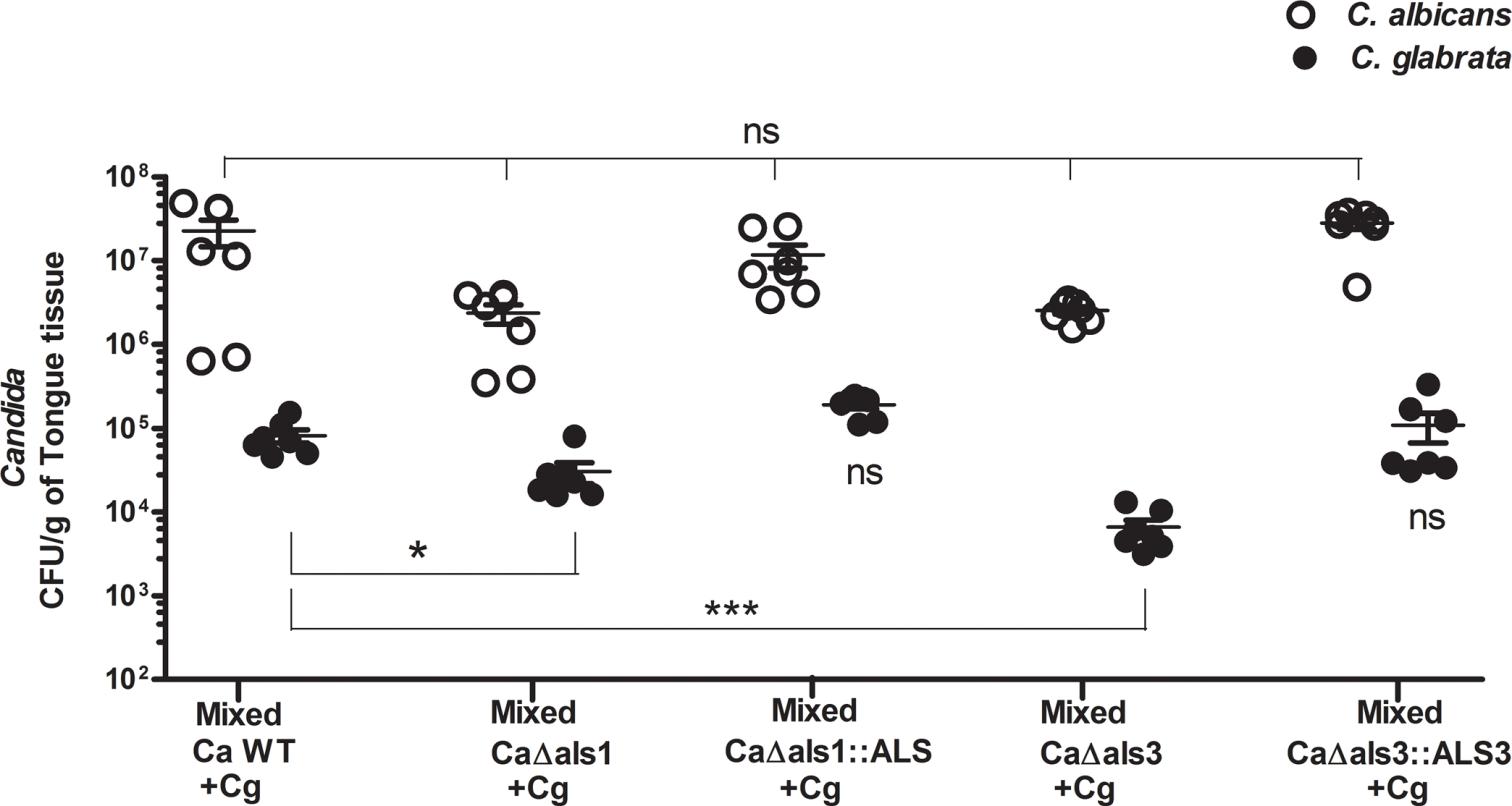
C. glabrata colonization requires C. albicans Als1 and Als3 in murine oropharyngeal candidiasis. Infection levels of Als1 and Als3 deficient strains of *C*. *albicans* did not differ significantly from WT; however, *C*. *glabrata* infection levels were decreased significantly in mice upon co-infection with *Ca als1Δ/Δ*, *als3Δ/Δ* mutants compared to WT. Mixed infection of *C*. *glabrata* with *C*. *albicans* Als1 and Als3 complementation strains showed restoration of *C*. *glabrata* colonization. Differences between groups were analyzed by a student’s t test (*P<0.02, **P<0.001).

Since our *in vitro* data showed that *C*. *albicans* Als1 and Als3 adhesins were important for *C*. *glabrata* adherence, we next examined their role in mixed *C*. *glabrata-C*. *albicans* oral infection *in vivo*. A 48 h delayed infection of *C*. *glabrata* following infection with *C*. *albicans* wild type or Als adhesin deficient strains was performed (Fig 7). *C*. *albicans als1Δ/Δ* and *als3Δ/Δ* mutants were able to establish infection at the same levels as WT cells. However, *C*. *glabrata* tongue CFUs were significantly (P<0.05) decreased (2.8 X 10^4^ CFU/g) following infection with the *C*. *albicans als1Δ/Δ* mutant; and were even further reduced (6.6 X 10^3^ CFU/g, P< 0.001) following infection by *C*. *albicans als3Δ/Δ*. Infection of *C*. *glabrata* with *C*. *albicans Als1* and *Als3* complemented strains showed restoration of *C*. *glabrata* colonization to levels similar to those observed with the wildtype *C*. *albicans* strain (Fig 7). No differences in animal weights between the groups was found since levels of infection by *C*. *albicans* were similar between groups.

### *C*. *glabrata* cell wall proteins are required for *C*. *albicans* adherence

**Fig 8:**
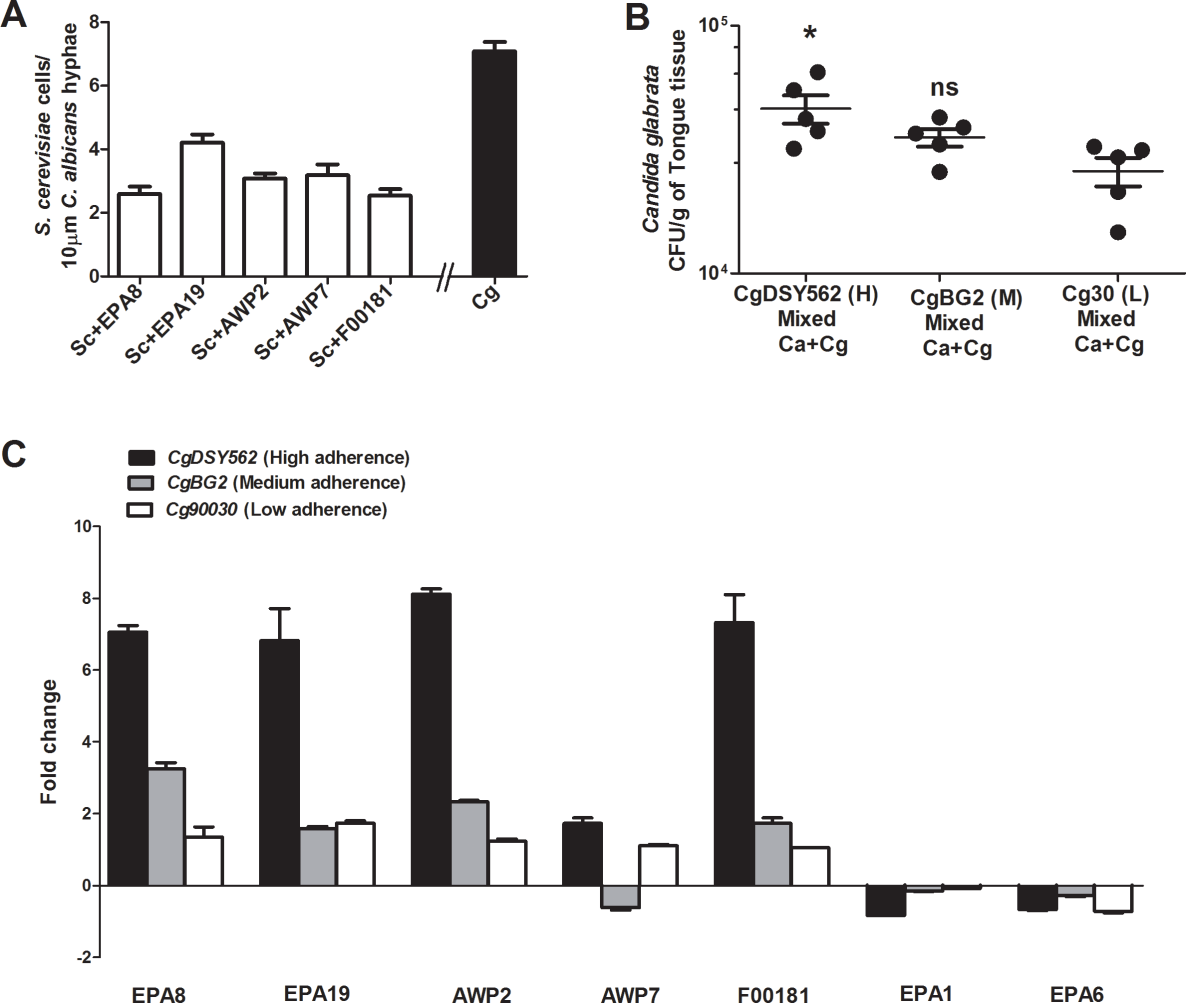
*C*. *glabrata* cell wall adhesins are induced in the presence of *C*. *albicans*. (**A**) Adherence of *S*. *cerevisiae* strains expressing *C*. *glabrata* cell wall adhesins to *C*. *albicans* hyphae was measured. Among 44 strains tested, only five strains were adherent to *C*. *albicans* hyphae, and adhesion levels were about half that of *CgDSY562* (Cg) (**B**) Mixed species infection with *CgDSY562* (High adherence), *CgBG2* (Medium adherence) and *Cg90030* (Low adherence) and *C*. *albicans CAI4* showed significant reduction in infection levels (*P<0.01 student’s t test) between the High (*CgDSY562*), and Low (*Cg90030*) adherence *C*. *glabrata* strains. (**C**) Expression levels of *C*. *glabrata EPA8*, *EPA19*, *AWP2*, *AWP7* and *CAGL0F00181* genes in three wild type strains *CgDSY562* (High adherence), *CgBG2* (Medium adherence) and *Cg90030* (Low adherence) were measured by qRT-PCR following incubation with germinated *C*. *albicans*. All five genes identified by screening (**A**) were induced in the presence of *C*. *albicans* hyphae proportionally with strain adherence and infection levels (**B**). In contrast, control *C*. *glabrata EPA1* and *EPA6* genes (far right) were not induced by *C*. *albicans* hyphae. The values plotted are means ± SEMs of n = 3 or 4 independent experiments.

To identify adhesion partners on *C*. *glabrata*, we screened 44 *S*. *cerevisiae* strains expressing *C*. *glabrata* cell wall proteins and identified five strains expressing *Cg*Epa8, *Cg*Epa19, *Cg*Awp2, *Cg*Awp7 or ORF *CAGL0F00181* that were most adhesive (2–5 cells/10 μm *C*. *albicans* hyphae) (Fig 8A). Most other tested strains, including the *S*. *cerevisiae* parental strain, had no adhesion to *C*. *albicans* hyphae. Next, we examined comparative transcription levels of these five candidate genes in *C*. *glabrata* strains which have high adherence (*CgDSY562*), medium adherence (*CgBG2*), and low adherance (*Cg90030*) *in vitro* to *C*. *albicans*. We used *C*. *glabrata EPA1* and *EPA6* genes as a negative control since *S*. *cerevisiae* expressing *C*. *glabrata* Epa1 and Epa6 did not bind to *C*. *albicans* hyphae, although they are highly expressed major adhesins in *C*. *glabrata*. To confirm that these strains also had differential binding to *C*. *albicans* during infection, we compared infection levels in a mixed infection in OPC, and found that indeed, the low and high adherence strains had a significant (P<0.01) difference in infection levels (Fig 8B). Then, transcriptional levels of these candidate genes were measured by qPCR before and after incubation with germinated *C*. *albicans*. Although *CgEPA8* and *CgAWP7* were most highly expressed in the high adhesion strain compared to the lower adhesion strains, we did not find significant differences in basal expression levels among the three other candidate genes among the *C*. *glabrata* strains. However, transcriptional levels of four genes (*CgEPA8*, *CgEPA19*, *CgAWP2*, and ORF *CAGL0F00181*) were increased significantly by 6–7 fold, while *CgAWP7* was increased by 2-fold in the high adherence strain (*CgDSY562*) upon incubation with *C*. *albicans* hyphae. This induction was less for *CgEPA19*, *CgAWP2*, and *CgCAGL0F00181* in the intermediate adherent strain, while the low adhesion *Cg90030* strain had the least induction by *C*. *albicans* for all five genes (Fig 8C). Expression of *CgEPA6* and *CgEPA1* genes, which serve as controls since they do not mediate adherence to *C*. *albicans*, were both modestly down-regulated in the presence of *C*. *albicans*. Taken together, these results show that *C*. *glabrata* cell wall genes *EPA8*, *EPA19*, *AWP2*, *AWP7* and *CAGL0F0018* are upregulated by *C*. *albicans* and may promote a dual species oral infection.

## Discussion

Although clinical studies have shown that *C*. *albicans* and *C*. *glabrata* are common partners co-isolated from oral infections, *C*. *glabrata* alone rarely causes oral infection. This work identifies for the first time that *C*. *glabrata* adherence to *C*. *albicans* hyphae is the basis for this partnership and that it is mediated by specific adhesins on both species. Previous *in vitro* studies found that *C*. *glabrata* alone was unable to colonize or invade reconstituted human vaginal epithelium (RHVE) [30] or reconstituted human oral epithelium (RHOE) [31]. Mixed infections using both *C*. *glabrata* and *C*. *albicans* increased tissue damage in RHOE [32] and were permissive for infection in RHVE [33] and *in vivo* in tongues of immunosuppressed mice [34], although others found no difference in host damage or inflammation in co-infected human oral epithelial [35]. These and our own studies are in agreement that *C*. *glabrata* alone is non-invasive in respect to oral-esophageal mucosal epithelium, in contrast to its ability to penetrate gastric epithelium [34]. The basis for this difference in tissue tropism is unknown, although it is possible that differences in the gut environment induces differential expression of *C*. *glabrata* adhesins.

We found that two major fungal hyphal wall adhesins Als3 and Als1 contribute to binding *C*. *glabrata in vitro* and to establish oral infection *in vivo*. *C*. *albicans* Als3 appears to make the major contribution towards binding with *C*. *glabrata*, with Als1 having a secondary role. Consistent with this, loss of Als1 on its own does not strongly reduce adherence to *C*. *glabrata*. However, in strains deleted for *ALS3*, additional loss of *ALS1* further reduced adherence by an additional two-fold. Als3 is a well known multifunctional surface protein, however we have identified an additional novel function of this adhesin in binding *C*. *glabrata*. Since Als 3 proteins are very abundant on *C*. *albicans* hyphae, and we only find 2–6 *C*. *glabrata* cells per hyphae, we expect that substantial numbers of Als proteins would still be available on hyphae to carry out other functions in the context of oral infection. It is also possible that Als3 might have a similar role in binding other non-hyphal forming *Candida* species such as *C*. *krusei* that are frequently co-isolated along with *C*. *albicans* in OPC. *C*. *albicans* Als3 seems to be promiscuous in its binding partners since Als3 proteins have been shown to bind the oral bacteria *S*. *gordonii* through its SspB cell surface protein in a mixed species biofilm [36] and to *S*. *aureus* during polymicrobial biofilm growth [37]. Hence Als3 may to be an excellent target for disruption of mixed species and inter-kingdom biofilms.

The *C*. *glabrata* Epa family consists of at least 20 GPI-anchored surface exposed adhesins whose expression of individual members is strain dependent [38]. Epa proteins recognize host glycans, and *C*. *glabrata* Epa1 is the best characterized member that is involved in adhesion to mammalian epithelium. Epa1 preferentially recognizes Gal β1–3 glycans, and variations of its adhesion domain conferred promiscuity of ligand binding [39]. Recently, Epa binding domains were functionally classified according to their ligand binding profiles, and interestingly our identified adhesins *C*. *glabrata* Epa8 and Epa19 were found to be very closely related and within the functional class III of Epa ligands that have weak binding to epithelial cells [40]. Thus, we speculate that some Class III Epa adhesins may have ligand functions with other cell types including *C*. *albicans*.

Another similarlity among the *C*. *glabrata* adhesins we identified (*EPA8*, *EPA19*, *AWP2*, *AWP7* and *CAGL0F00181*) is that their expression levels were all induced by incubation with *C*. *albicans* hyphae (Fig 8C). In contrast, *C*. *glabrata EPA1* and *EPA6* (both Class I ligands with high binding to epithelial and endothelial [41] cells, and highly expressed in log phase cells [23]), were not up-regulated following incubation with *C*. *albicans* hyphae. In agreement with our findings, no increase in expression levels of *C*. *glabrata EPA1*, *EPA6* or *EPA7* was found following co-infection with *C*. *albicans* in RHVE cells [30]. Based on our results, we propose a role of these *C*. *glabrata* CWPs (*EPA8*, *EPA19*, *AWP2*, *AWP7*, and *CAGL0F00181)* in interspecies binding and further suggest that *C*. *glabrata* is able to transcriptionally regulate selected genes needed for its colonization and survival in a host. It is known that many *C*. *glabrata EPA* genes are transcriptional silenced. Since *EPA1* and *EPA6* (both of which are strongly silenced) are not up-regulated by co-culture with *C*. *albicans* (Fig 8), this suggests that the transcriptional regulation of *C*. *glabrata EPA8*, *EPA19*, *AWP2*, *AWP7*, and *CAGL0F00181* is not through general antagonism of sub-telomeric silencing [42]. How *C*. *glabrata* regulates these genes in the presence of *C*. *albicans* remains to be determined.

*C*. *glabrata* alone was not competent to cause infection in our OPC model. Our data further suggest that while *C*. *glabrata* colonizes oral mucosa poorly (even in an immunosuppressed host), it has evolved to exploit the presence of hyphae-producing *C*. *albicans* to establish colonization and invasion of oral epithelium; and its presence enhanced the severity of OPC as measured by rate of weight loss of animals. Furthermore, co-infections treated with Fluconazole reduced levels of *C*. *glabrata* concomitantly with *C*. *albicans* over four days, showing its dependence upon the presence of *C*. *albicans* in early infections. However, our results show that *C*. *glabrata* is found both together and apart from *C*. *albicans* hyphae in tissues, suggesting that once it gains a foothold in oral epithelium by binding *C*. *albicans* hyphae, it can survive alone in mucosal tissues, albeit at low levels. These *C*. *glabrata* cells existing independently in oral mucosa may be a colonization reservoir for dissemination if the oral epithelium is breached by trauma, chemotherapy or other factors. In this regard, our preliminary experiments showed that mice with mixed *C*. *glabrata* and *C*. *albicans* oral infections had significantly higher stomach colonization of both species, suggesting that gut colonization might serve as such a reservoir. Also, these reservoirs may become clinically significant following long-term azole therapy providing an environment in which drug resistant *C*. *glabrata* could emerge.

Our data suggests a model whereby oral tissues that are inherently resistant to infection by *C*. *glabrata*, are colonized by piggybacking with *C*. *albicans* to establish a foothold of tissue infection. Of interest, and the subject of ongoing studies in our lab, is the role of oral and gut reservoirs of *C*. *glabrata* in subequent colonization of other tissues that have a naturally higher tropism for infection by *C*. *glabrata*, as well as their role in subsequent dessimination.

## Methods

### Strains

All *Candida* and *S*. *cerevisiae* strains used are listed in Table 1 and Table in S1 Table. *C*. *albicans* cells were maintained in yeast extract/peptone/dextrose (YPD; Difco) medium with the addition of uridine (50 mg/ml; Sigma) when required and stored as -80°C. *S*. *cerevisiae* containing pADH or pADH-ALS3 were maintained on synthetic medium lacking uracil (CSM-glu) (0.077% CSM-ura, 0.67% yeast nitrogen base [Difco], 1.25% glucose, and 2.5% agar). *S cerevisiae* strains expressing N-terminal domains of *C*. *glabrata* Cell Wall Proteins (CWP) were made as described [42], and are in preparation for publication elsewhere). The ORFs whose domains mediate adherence to *Candida* hyphae are shown in S1 Table.

**Table 1:** List of strains used in this study.

Strain	Relevant Genotype	*URA* status	Reference
***C*. *albicans***			
*CAI4+URA*	*Δura3*::*imm434/Δura3*::*imm434RPS1/Δrps1*::*Clp10-URA3*	+	[49]
*DAY185*	*ura3Δ*::*λimm434/ura3Δ*::*λimm434arg4*::*hisG*::*ARG4*::*URA3/arg4*::*hisG*	+	[50]
	*his1*::*hisG*::*HIS1/his1*::*hisG*		
*CAF2-yCherry*	*ura3*Δ::*imm434/URA3 PADH1-yCherry_NATR*	+	[45]
*DSY294*	*Azole-susceptible clinical strain*	+	[51]
*DSY296*	*Azole resistant clinical strain*	+	[51]
*CAYF178U*	*ura3*::*imm434*::*URA3-IRO1/ura3*::*imm434arg4*::*hisG/arg4*::*hisG/his1*::*hisG/his1*::*hisG als3*::*ARG4/als3*::*HIS1*	+	[52]
*CAQTP178U*	*ura3D*:: *λkimm434*::*URA3-IRO1 als3*::*ARG4*::*ALS3 arg4*::*hisG his1*::*hisG*	+	[52]
	*ura3D*:: *λkimm434 als3*::*HIS1 arg4*::*hisG his1*::*hisG*		
*CAYC2YF1U*	*ura3D*: *λkimm434*::*URA3-IRO1 als1*::*hisG /ura3D*::*imm434 als1*::*hisG*	+	[53]
*CAYC1*	*ura3D*:: *λimm434/ura3D*::*imm434*, *als1D*::*hisG-URA3-hisG/ALS1*	+	[53]
*CJN1348*	*ura3Δ*::*λimm434*:: *URA3-IRO1 als1*::*hisG als3*::*dpl200*	+	[54]
	*ura3Δ*::*λimm434 als1*::*hisG als3*::*dpl200*		
*CJN1352*	*ura3Δ*::*λimm434*::*ALS1*::*URA3-IRO1 als1*::*hisG als3*::*dpl200*	+	[54]
	*ura3Δ*::*λimm434 als1*::*hisG als3*::*dpl200*		
*CJN1356*	*ura3Δ*::*λimm434*::*ALS3*:: *URA3-IRO1 als1*::*hisG als3*::*dpl200*	+	[54]
	*ura3Δ*::*λimm434 als1*::*hisG als3*::*dpl200*		
***S*. *cerevisiae***			
*S150-2B pADH1*	*leu2 his3 trp1 ura3+pADH1*	-	[55]
*S150-2B pALS3*	*leu2 his3 trp1 ura3+pALS3*	-	[55]
*S150-2B pALS1*	*leu2 his3 trp1 ura3+pALS1*	-	[55]
***C*. *glabrata***			
*BG2*	*Wild type*	+	ATCC
*DSY562*	*Azole-susceptible clinical strain*	+	[56]
*DSY565*	*Azole resistant clinical strain*	+	[56]
*VSY55*	*DSY562 ura3*::*hph*, *ScPGK1p-yEGFP*, *URA3*, *CEN-ARS*	+	[46]
*Cg90030*	*Wild type*	+	ATCC
*Cg60032*	*Wild type*	+	ATCC
*Cg931010*	*Wild type*	+	[48]
*Cg932474*	*Wild type*	+	[48]
*Cg148042*	*Wild type*	+	ATCC

### Adhesion assays

*C*. *albicans* cells were cultured overnight in YPD broth, diluted to an OD_600_ = 0.3 in pre-warmed YNB medium supplemented with 1.25% GlcNAc, and incubated for 3 h at 37°C with gentle shaking to induce germination. *C*. *glabrata* or *S*. *cerevisiae* strains were grown similarly except in YNB + 1.25% of glucose. Cells were collected by centrifugation (100 X g), washed once in PBS, and then re-suspended in PBS. Germination of *C*. *albicans* cells was confirmed by microscopic observation. *C*. *albicans* cells were then incubated with *C*. *glabrata* cells at a 1:1 ratio for 60 min. Blocking experiments described previously [29], were carried out using washed *C*. *albicans* cells incubated with concanavalin A (100 ug/ml; mannan binding lectin, Sigma) or β,1–3 glucan Ab (10 μg/ml, Biosupplies) for 30 min (concentrations that gave high coverage of cells as determined by FACScan), then washed in PBS before assay. For adhesion assays of *S*. *cerevisiae* strains expressing *C*. *albicans* Als1 and Als3 adhesins, *S*. *cerevisiae* cells or an *S*. *cerevisiae* empty vector (control) were incubated with *CgVSY55* for 1 h at 37°C (at cell ratio 1:1), then a Binding Index was calculated as the number of *C*. *glabrata* cells bound to *S*. *cerevisiae* cells divided by (number of bound *C*. *glabrata* cells plus unbound *C*. *glabrata* cells plus unbound *S*. *cerevisiae* cells) X 100 per field. At least 10 separate fields were used to obtain averages.

### Biofilm assays

Each *Candida* strain was grown overnight to OD_600_~2.0, washed twice in Phosphate Buffered Saline (PBS), re-suspended in YNB without uridine, and 1 ml cells (1 X10^6^ cells/ml) were added to polystyrene wells. For mixed species biofilms, 500 μl of each species (5 X 10^5^ cells /ml) for a total of 1 ml was added to the well. After incubation for 3 h to allow adhesion, non-adherent cells were gently removed by aspiration and 1 ml of fresh media was added. Biofilms were grown for 24 h at 37°C on an orbital shaker and biofilm dry weight was measured as previously described [43]. For fluorescence biofilm assays, single and dual species biofilms were grown on 96 well microtiter plates using a yCherry expressing strain of *C*. *albicans* and a GFP expressing *C*. *glabrata* strain. Fluorescent counts were recorded at 37°C using a Bio-Tek multifunction plate reader and analyzed using Gen5 software. Alternatively, we examined non-static dual species biofilms grown under flow conditions. For these experiments, YPD media containing the *C*. *albicans* WT strain CAF2 cells expressing the fluorescent protein mCherry and the *C*. *glabrata* WT strain VSY55 expressing GFP (both at 1 × 10^6^ cells/ml) were circulated through a μ-Slide I 0.8 Luer family ibiTreat flow chamber (ibidi, Martinsried, Germany) for 2 h at 37°C and a shear force at the coverslip surface of 0.8 dynes/cm^2^. Images were obtained using a Zeiss LSM 510 confocal microscope, and analyzed using ZEN imaging software (Zeiss, Göttingen, Germany). Flow was maintained during image acquisition.

### RNA extraction and qRT-PCR

Overnight cultures of *C*. *albicans* were diluted to an OD_600_ = 0.3 in pre-warmed YNB medium supplemented with 1.25% GlcNAc and incubated for 3 h at 37°C to induce germination, or diluted in YNB medium supplemented with 1.25% glucose at room temperature for yeast cells. *C*. *glabrata CgDSY562*, *CgBG2*, and *Cg90030* overnight cultures were grown similarly using YNB + 1.25% of glucose. Cells were collected by centrifugation (100 X g), and re-suspended in PBS. *C*. *glabrata* cells were then incubated with germinated or yeast form *C*. *albicans* at a 1:1 ratio for 30 min. Total RNA was isolated from *C*. *glabrata*, *C*. *glabrata* and *C*. *albicans* 1 X 10^7^ cells using an RNeasy minikit (Qiagen). Reverse transcription (RT) was performed using SuperScript III reverse transcriptase, and oligo(dT)20 primer (Invitrogen). cDNA was purified (Geneflow PCR purification kit) and quantified with a NanoDrop spectrophotometer (NanoDrop Technologies). Quantitative RT-PCR (qRT-PCR) was performed in triplicate for *CgACT1*, *CgAWP2*,*7*, *CgEPA1*,*6*,*8*,*19* and *CgCAGL0F00181* using gene-specific primers (Table in S2 Table). *C*. *albicans* cDNA was used as a negative control in all experiments to verify specifity of amplification. Genes were normalized to *CgACT1* in each respective strain and condition as decribed previously [44].

### Microscopy

Fluorescent microscopy was done using a yCherry expressing strain of *C*. *albicans* [45] and the GFP expressing *C*. *glabrata* (*VSY55*: *ura3Δ*::*hph* ScPGKp-yEGFP-URA3-CEN-ARS) derived from a C. *glabrata DSY562* clinical isolate [46]. Scanning electron microscope observations were carried out on *C*. *albicans* hyphae and *C*. *glabrata* cells. *C*. *albicans* cells were grown in YNB for yeast phase cells or in YNB + 1.25% GlcNAc at 37°C (to induce hyphae) for 3 h. *C*. *albicans* cells were then incubated with *C*. *glabrata* cells at 1:1 ratio for 30 min. Cells were incubated on a concavalin A (100ug/ml; Sigma) coated glass slide for 1 h at RT. Cells were washed twice with PBS, fixed with 2% glutaraldehyde (Sigma) for 30 min at 4°C, then washed twice with distilled water. Samples were dehydrated in 30%, 50%, 70%, 85%, and 95% ethanol for 15 min each and 100% ethanol twice for 15 min each. Samples were exchanged into 100% hexamethyldisilazane (HMDS) and allowed to dry in a hood before visualization. SEM observation was done under the following analytical condition: L = SE1 and EHT = 2.5 kV to study the binding of *C*. *glabrata* on *C*. *albicans* cells with Hitachi SU70 FESEM operating at 2.0 keV.

### Mixed Candida infection in murine oral candidiasis

*C*. *albicans* murine OPC model [47,48] was used for infection with *C*. *glabrata*. Mice (BALB/c, C57BL/6, and IL17RAk/o) were immuno-suppressed with cortisone acetate (150–250 mg/kg), triampicinolone acetonide (100–150 mg/kg) or cyclophosphamide (100–150 mg/kg) one day before infection with *C*. *glabrata* (1 X 10^7^ to 1 X 10^9^ cells/ml). For mixed infections, mice (female C57BL/6, 4–6 weeks old) were immunosuppressed with cortisone acetate 225 mg/kg (Sigma) on day -1, +1, and +3, and then infected with *C*. *albicans* (5 X 10^7^ cells/ml) on day 0; or infected with *C*. *glabrata*, (1 X 10^9^ cells/ml) on day 2 after pre-establishing *C*. *albicans* infection on day 0. *C*. *albicans* and *C*. *glabrata* colonies from tongue tissues were differentiated on CHROMagar media. On the fifth or sixth day after infection, mice were euthanized by cervical dislocation under anesthesia (ketamine/xylazine); tongue tissues were excised and hemi-sectioned along the long axis with a scalpel. One half was weighed and homogenized for quantification of fungi, and the other half was processed for histopathological analysis. Tongue hemi-sections were fixed in 10% buffered-formalin for 24 h, paraffin embedded, and then cut into 5μm sections for Periodic Acid-Schiff (PAS) staining as we previously described [49]. For histological co-localization experiments, animals were infected with *C*. *albicans* yCherry and the GFP expressing *C*. *glabrata* (*VSY55*) strains as described above. For these experiments, tongue hemi sections were fixed in 4% (w/v) paraformaldehyde (PFA) for 24 h, incubated in 30% sucrose for 3 days, snap frozen in OCT compound (Tissue-Tek, Sakura, Torrance, CA) with liquid nitrogen, and cut into 8μm cryosections.

For Fluconazole (Flu) treatment studies, ten mice were used for each group (drug treatment and controls using combinations of Flu resistant and sensitive strains of *C*. *albicans* and Flu resistant *C*. *glabrata* shown in Table 1). Sensitivities of each strain to Flu was verified using MIC assays. Immunosuppression was induced on days −1, +1 and +3 post-infection. Mice were infected sublingually with *C*. *albicans* (5 X 10^7^ cells/ml) on day 0, and *C*. *glabrata* (1 X 10^9^ cells/ml) on day 2, and were sacrificed on day +7. Mice received daily intraperitoneal injections of 100 mg/kg Fluconazole that was initiated 48 h after *C*. *glabrata* infection and continued through post-infection day 7.

### Statistics

Statistical analyses were performed using GraphPad Prism software version 5.0 (GraphPad Software, San Diego, CA, USA) using unpaired Student's t-tests. Differences of P<0.05 were considered significant. All experiments were performed at least thrice.

### Ethics statement

This study was carried out in strict accordance with the recommendations in the Guide for the Care and Use of Laboratory Animals of the National Institutes of Health. This protocol was approved by the University of Buffalo Institutional Animal Care and Use Committee (Project Number: ORB06042Y).

## Supporting Information

S1 Table List of *S*. *cerevisiae* strains expressing *C*. *glabrata* adhesins. (DOCX)

S2 Table List of *C*. *glabrata* genes and qRT-PCR primer sequences. (DOCX)
