# Supplementary material for: Candida glabrata Binding to Candida albicans Hyphae Enables Its Development in Oropharyngeal Candidiasis
Source: PLoS Pathog. 2016 Mar 30;12(3):e1005522. doi: 10.1371/journal.ppat.1005522 (PMC4814137; doi:10.1371/journal.ppat.1005522)
Supplement: S1 Table — (DOCX) [file ppat.1005522.s001.docx]

**S1 Table. *S. cerevisiae* strains expressing *C. glabrata* adhesins**

| **Strains** | ***C. glabrata* ORF insert** | ***URA* Status** | **Reference** |
| --- | --- | --- | --- |
| *SC608/SC609* | EPA24 (CAGL0A00162g) | + | This work |
| *SC119* | EPA19 (CAGL0A00159g) | + | This work |
| *SC591* | CAGL0B00159g | + | This work |
| *SC592* | CAGL0B05054g | + | This work |
| *SC612/SC613* | CAGL0C00269g | + | This work |
| *SC636/SC637* | CAGL0C00209g | + | This work |
| *SC33* | EPA6 (CAGL0C00110g) | + | This work |
| *SC610/SC611* | EPA25 (CAGL0C05595g) | + | This work |
| *SC580* | EPA26 (CAGL0C05620) | + | This work |
| *SC587* | CAGL0D00148g | + | This work |
| *SC120* | EPA21 (CAGL0D06715g) | + | This work |
| *SC626/SC627* | CAGL0E00269g | + | This work |
| *SC630/SC631* | CAGL0E00187g | + | This work |
| *SC35* | EPA7 (CAGL0C05604g) | + | This work |
| *SC632/SC633* | CAGL0E06600g | + | This work |
| *SC49* | EPA1 (CAGL0E06644g) | + | This work |
| *SC45* | EPA2 (CAGL0E06666g) | + | This work |
| *SC29* | EPA3 (CAGL0E06688g) | + | This work |
| *SC600/SC601* | CAGL0F00181g | + | This work |
| *SC588* | EPA16 (CAGL0F00170g) | + | This work |
| *C581* | CAGL0F09231g | + | This work |
| *SC618/SC619* | CAGL0E00181g | + | This work |
| *SC602/SC603* | CAGL0G10175g | + | This work |
| *SC582* | CAGL0G10180g | + | This work |
| *SC589* | CAGL0H00214g | + | This work |
| *SC121* | EPA23 (CAGL0I00220g) | + | This work |
| *SC583* | CAGL0I00214g | + | This work |
| *SC47* | EPA4 (CAGL0I11000g) | + | This work |
| *SC31* | EPA5 (CAGL0I10992g) | + | This work |
| *SC616/SC617* | CAGL0I10995g | + | This work |
| *SC628/SC629* | AWP3a (CAGL0J11885g) | + | This work |
| *SC614/SC615* | AWP3b (CAGL0J11896g) | + | This work |
| *SC229* | EPA15 (CAGL0J11968g) | + | This work |
| *SC590* | CAGL0J11973g | + | This work |
| *SC131* | EPA22 (CAGL0K00225g) | + | This work |
| *SC584* | AWP2 (CAGL0K00110g) | + | This work |
| *SC634/SC635* | CAGL0K13002g | + | This work |
| *SC585* | CAGL0K13007g | + | This work |
| *SC620/SC621* | CAGL0L00269g | + | This work |
| *SC41* | EPA11 (CAGL0L13311g) | + | This work |
| *SC43* | EPA13 (CAGL0L13333g) | + | This work |
| *SC51* | EPA12 (CAGL0M00132g) | + | This work |
| *SC586* | CAGL0F09234 | + | This work |
| *SC622/SC623* | PWP6 (CAGL0M14069g) | + | This work |

**S2 Table. *C. glabrata* genes and qRT-PCR primer sequences.**

| ***C. glabrata* genes** | **Primer sequences** |
| --- | --- |
| *CgACT1* | GACGGCGATTATGAGTTAGGAG  GTAGCATCTGTGCAGGTAGTT |
| *CgEPA1* | GGGCTCAAAAACAGCTAAG  TAACAGTTGTTTTCGTTTGAT |
| *CgEPA6* | GAAATCAGGATCGAATCCATG  GTGGTAATGTATCAAACAGCG |
| *CgEPA8* | CAGGTGATCCAGAAAGTCCAA  CAGTCGTGGTGATAGTTGTAG |
| *CgEPA19* | GCAGGCAGTAATGTACCATAT  GAGTGTGGTGTTTGGCTACT |
| *CgAWP2* | CCAAGGTAGCTCTTTGGAGATG  TGGAAGATGGAGGCAGTTTG |
| *CgAWP7* | CCACTTCTGCTTCCTCTTCTAC  AGGGACTACTCGAAGCTGATA |
| *CAGL0F00181* | CAACCCTACTGGGTATGATG  ACAAATAAAGCGTGGCTAGA |
