# Supplementary material for: Candida glabrata Binding to Candida albicans Hyphae Enables Its Development in Oropharyngeal Candidiasis
Source: PLoS Pathog. 2016 Mar 30;12(3):e1005522. doi: 10.1371/journal.ppat.1005522 (PMC4814137; doi:10.1371/journal.ppat.1005522)
Supplement: S2 Table — (DOCX) [file ppat.1005522.s002.docx]

**S2 Table. *C. glabrata* genes and qRT-PCR primer sequences.**

| ***C. glabrata* genes** | **Primer sequences** |
| --- | --- |
| *CgACT1* | GACGGCGATTATGAGTTAGGAG  GTAGCATCTGTGCAGGTAGTT |
| *CgEPA1* | GGGCTCAAAAACAGCTAAG  TAACAGTTGTTTTCGTTTGAT |
| *CgEPA6* | GAAATCAGGATCGAATCCATG  GTGGTAATGTATCAAACAGCG |
| *CgEPA8* | CAGGTGATCCAGAAAGTCCAA  CAGTCGTGGTGATAGTTGTAG |
| *CgEPA19* | GCAGGCAGTAATGTACCATAT  GAGTGTGGTGTTTGGCTACT |
| *CgAWP2* | CCAAGGTAGCTCTTTGGAGATG  TGGAAGATGGAGGCAGTTTG |
| *CgAWP7* | CCACTTCTGCTTCCTCTTCTAC  AGGGACTACTCGAAGCTGATA |
| *CAGL0F00181* | CAACCCTACTGGGTATGATG  ACAAATAAAGCGTGGCTAGA |
